# Primary report for a randomized controlled trial of traumatic spinal cord injured patients from T1 to L1 - description of the surgical decompression in two groups of before 24 hours and 24 to 72 hours

**Published:** 2012-11

**Authors:** Vafa Rahimi-Movaghar, Ali Haghnegahdar, Amin Niakan, Aidin Omidvar, Ehsan Barzideh, Fahim Baghban, Mohammad Jamali, Navideh Mohebali, Hamed Yazdanpanah, Seyede Maryam Fallahi, Mehdi Salimi Sotoudeh, Mohammad Reza Sharifirad

**Affiliations:** ^*a*^Sina Trauma and Surgery Research Center, Tehran University of Medical Sciences, Tehran, Iran.

## Abstract

**Background:**

There is no clear evidence that early decompression following spinal cord injury (SCI) improves neurologic outcome. In this primary report for prospective, randomized clinical trial, 35 selected spinal cord injured patients with traumatic thoracolumbar spinal cord injury were randomly assigned to early surgery (before 24 hours); and late surgery (24–72 hours).

**Methods:**

Seventeen patients were assigned to the early and 18 to the late surgery. Twenty-five patients (71.4%) were male. Mean age of patients was 34 ± 12 years old. The most common levels of SCI were L1, T12, and T11 in 34%, 29%, and 11%, respectively. Sixteen (62.5%) had complete SCI (American Spinal Injury Association Impaired Scale (AIS) A. Number of patients with AIS B, C, D and E were 6, 5, 4, and 4, respectively. Follow-up of patients showed AIS A, B, C, D, and E in 7, 12, 4, 5, and 6 patients, respectively.

One patient (3%) was deteriorated who was from the early surgery group. No change in neurologic deficit was seen in 12 patients (34%). Eighteen patients (52%) improved one AIS grade, 8 were early and 10 late surgery. Three patients (9%) improved two AIS grades all were early surgery. Not available follow-up data for one patient (3%).

**Results:**

Only 3/7 patients with AIS A in early surgery had one AIS grade improvement. In late surgery, 6/9 patients with AIS A had just one AIS grade improvement. Mean duration of hospitalization for all SCI patients were 11 ± 10 days, which was 8 ± 8 days for early and 14±12 days for late surgery.

**Conclusions:**

Complications were two deaths, one in early surgery because of pulmonary emboli. Second death was in late surgery with unknown etiology. Two cases had deep vein thrombosis in early surgery. In late surgery, three cases had cerebrospinal fluid leak, meningitis and wound infection. Number of patients was not enough for comparing two surgery groups. However, both early and late surgery groups had some improvement in almost half of SCI patients.

**Keywords:**

Randomized controlled trial, Traumatic spinal cord injury, Thoracolumbar, Surgical decompression, Time


					Volume 4, Suppl. 1 Nov 2012
Publisher: Kermanshah University of Medical Sciences
URL: http://www.jivresearch.org
Copyright:
Congress of Iranian Neurosurgeons, 14-16 November 2012, Kermanshah, Iran
Paper No. 27
Primary report for a randomized controlled trial of traumatic
spinal cord injured patients from T1 to L1 - description of the
surgical decompression in two groups of before 24 hours and
24 to 72 hours
Vafa Rahimi-Movaghara,*
, Ali Haghnegahdar a
, Amin Niakan a
, Aidin Omidvar a
, Ehsan Barzideh a
,
Fahim Baghban a
, Mohammad Jamali a
, Navideh Mohebali a
, Hamed Yazdanpanah a
,
Seyede Maryam Fallahi a
, Mehdi Salimi Sotoudeh a
, Mohammad Reza Sharifirad a
a
Sina Trauma and Surgery Research Center, Tehran University of Medical Sciences, Tehran, Iran.
Abstract:
Background: There is no clear evidence that early decompression following spinal cord injury (SCI) improves
neurologic outcome.
In this primary report for prospective, randomized clinical trial, 35 selected spinal cord injured patients with
traumatic thoracolumbar spinal cord injury were randomly assigned to early surgery (before 24 hours); and late
surgery (24-72 hours).
Methods: Seventeen patients were assigned to the early and 18 to the late surgery. Twenty-five patients (71.4%)
were male. Mean age of patients was 34  12 years old. The most common levels of SCI were L1, T12, and T11 in
34%, 29%, and 11%, respectively. Sixteen (62.5%) had complete SCI (American Spinal Injury Association Impaired
Scale (AIS) A. Number of patients with AIS B, C, D and E were 6, 5, 4, and 4, respectively. Follow-up of patients
showed AIS A, B, C, D, and E in 7, 12, 4, 5, and 6 patients, respectively.
One patient (3%) was deteriorated who was from the early surgery group. No change in neurologic deficit was seen
in 12 patients (34%). Eighteen patients (52%) improved one AIS grade, 8 were early and 10 late surgery. Three
patients (9%) improved two AIS grades all were early surgery. Not available follow-up data for one patient (3%).
Results: Only 3/7 patients with AIS A in early surgery had one AIS grade improvement. In late surgery, 6/9
patients with AIS A had just one AIS grade improvement. Mean duration of hospitalization for all SCI patients were
11  10 days, which was 8  8 days for early and 1412 days for late surgery.
Conclusion: Complications were two deaths, one in early surgery because of pulmonary emboli. Second death was
in late surgery with unknown etiology. Two cases had deep vein thrombosis in early surgery. In late surgery, three
cases had cerebrospinal fluid leak, meningitis and wound infection. Number of patients was not enough for
comparing two surgery groups. However, both early and late surgery groups had some improvement in almost half
of SCI patients.
Keywords:
Randomized controlled trial, Traumatic spinal cord injury, Thoracolumbar, Surgical
decompression, Time
* Corresponding Author at:
Vafa Rahimi-Movaghar: Associate professor of Neurosurgery, Research Deputy, Sina Trauma and Surgery Research Center, Sina Hospital,
Hassan-Abad Square, Imam Khomeini Ave, Tehran University of Medical Sciences, Tehran, Iran. Phone: (+98) 915 342 2682, (+98) 216 6757010
Fax: (+98) 216 675 7009, Email: v_rahimi@sina.tums.ac.ir , v_rahimi@yahoo.com, (Rahimi-Movaghar V.).

				

